# Effect of Oxidization Temperatures and Aging on Performance of Carbonate Melt Oxidized Iridium Oxide pH Electrode

**DOI:** 10.3390/s19214756

**Published:** 2019-11-01

**Authors:** Penggang Wang, Tengfei Guo, Tiejun Zhao, Zhenxing Du, Zuquan Jin, Biqin Dong, Zhe Li

**Affiliations:** 1 School of Civil Engineering, Qingdao University of Technology, Qingdao 266033, China; guotf127@163.com (T.G.); ztjgp@qut.edu.cn (T.Z.); 18354229660@163.com (Z.D.); jinzq@qut.edu.cn (Z.J.); lizhequt@163.com (Z.L.); 2 Guangdong Provincial Key Laboratory of Durability for Marine Civil Engineering, Shenzhen University, Shenzhen 518060, China; incise@szu.edu.cn; 3 Shenzhen Durability Center for Civil Engineering, Shenzhen University, Shenzhen 518060, China; 4 Cooperative Innovation Center of Engineering Construction and Safety in Shandong Blue Economic Zone, Qingdao 266033, China

**Keywords:** iridium oxide, pH electrode, carbonate melt oxidized, oxidation temperature, microstructure

## Abstract

Iridium oxide pH electrodes employing the carbonate melt oxidation method were fabricated with oxidation temperatures of 750 °C, 800 °C and 850 °C, respectively. Scanning electron microscope (SEM) and atomic force microscope (AFM) images showed that the oxide film regularized with the increase in oxidation temperatures. The pH response, response time and long-term stability of the electrodes indicated that the electrodes made at 850 °C had the best performance. X-ray photoelectron spectra (XPS) surveys investigated the change in the electrodes’ chemical composition and element oxidation states at 850 °C, and the results showed that the relative content of Ir^3+^ had increased by 23.9%, and the Ir^4+^ and Ir^6+^ had decreased by 10.9% and 13%, respectively, in the surface oxide layer after one month of aging. However, the relative contents of Ir^3+^, Ir^4+^ and Ir^6+^ were almost constant for the inner oxide layer. Meanwhile, the XPS result also indicated that the outer oxide layer of the electrode had a higher hydration degree than the inner oxide layer.

## 1. Introduction

In general, pH is one of the most important parameters which has to be measured in many works of analytical chemistry research. For example, carbonation, corrosion, and acid attack of reinforced concrete structure are related to the pH value of the pore solution [1,2,3,4]. The pH value of normal concrete is usually between 12.5 and 13 because calcium hydroxide is one of the major cement hydration products. However, it will decrease due to carbonation, chloride ingress, or acid attack. Normally, reinforcing steel in concrete does not corrode because a compact and stable passive film always forms on the surface of the reinforcing steel in the highly alkaline concrete pore solutions. Carbonation of calcium hydroxide in concrete can reduce the pH of concrete to values less than 9 [5,6,7,8]. When the pH value in vicinity of the rebar is lower than 11, initiation of active corrosion will happen even without chloride ions near rebar [9]. Therefore, in situ pH measurements of hardened concrete is very important for durability assessment and maintenance of reinforced concrete. Glass membrane electrodes are the most common electrodes for measuring the pH value of solutions [10]. However, glass electrodes are unsuitable for use in strong alkaline solutions, as well as in biomedical, concrete, and food process monitoring, because of the alkaline error. Additionally, it is difficult to miniaturize them, and their glass substrate is fragile. Therefore, many researchers have developed alternative electrodes. Metal oxide electrodes are considered suitable for overcoming the aforementioned problems, due to their stability and ruggedness, their prompt response and their flexibility in terms of size. The theoretical background of using the metal oxide electrodes for measuring pH, relies on reversibility of their redox reaction in aqueous solutions. Several metal oxide electrodes have been developed by different researchers [11,12,13,14,15,16,17,18,19], such as IrO_X_, PbO_2_, OsO_2_, TiO_2_, WO_3_, PtO_2_, Sb_2_O_3_, and RuO_2_. Among them, IrO_X_ is considered the most suitable material for pH electrodes used in cementitious material [11]. However, IrO_X_ electrodes are the most developed electrodes amongst them for its high stability within a wide pH range, fast response time, even in harsh conditions, such as high pressure and high temperatures, or in aggressive environments [12,20,21]. There are many methods for fabricating IrO_X_ electrodes for pH electrodes, including electrochemical growth [22,23], electrodeposition [24,25], sputtered coating [26,27], and thermal method [28]. However, the problem of electrode potential drift raised from these methods limits the widespread application of IrO_X_ pH electrodes. Yao et al. [11] have reported a method for making IrO_X_ electrodes by oxidizing the Ir substrate in molten carbonate. IrO_X_ electrodes made in this way have excellent pH response characteristics and can maintain stability for at least 2.5 years. However, problems pertaining to electrode potential drift and lack of long-term stability remains, even when using the carbonate melt oxidation method to prepare IrO_X_ electrodes [29,30,31]. Researchers have developed improved methods based on carbonate melt oxidation. For example, Pan et al. [30] used Li_2_CO_3_ and Na_2_O_2_ as the melt bath to oxidize iridium. The improved electrode has good reproducibility and a small drift potential. O’Malley et al. [31] have developed the vertical melt oxidation method which provides a more uniform reaction environment than previous methods. They also have investigated the effect of oxidation time on IrO_X_ electrodes. Chen et al. [32] have investigated the impact of carbonate types on IrO_X_ electrodes by using different carbonates as the oxidation media. Prior research has shown that the oxidation temperature, molten carbonate composition, oxidation time and hydration degree can influence the characteristics of IrO_X_ electrodes. The mechanism of metal oxide film growth in molten carbonate has been investigated in molten carbonate fuel cells, and the oxidizing specie is O_2_^2−^, which is produced from the reaction of dissolved O_2_ in melted CO_3_^2−^. Moreover, under the same partial pressure of carbon dioxide, the diffusion coefficient of O_2_^2−^ in molten carbonate increases with the increasing oxidation temperatures [33].

Since the oxidation temperature is an important factor in melt-oxidized IrO_X_ electrode fabrication, its influence on the electrode should not be ignored. However, the effect of oxidation temperatures on the performance of IrO_X_ pH electrodes has received insufficient investigation. The melt point of lithium carbonate is about 723 °C, the fabrication temperature of this method must over 723 °C. And higher temperature can benefit to the stability of the electrode, but it can also accelerate the decomposition rate of the lithium carbonate, thus lead to the insufficient oxidation of iridium and adversely affect the stability of the electrode. If the temperature up to 900 °C, the oxidation time must be varied. Effect of oxidation time on performance of carbonate melt oxidized Iridium oxide pH electrode well be mentioned in another paper. In this paper, the carbonate melt oxidation method was adopted to fabricate IrO_X_ electrodes, and three different oxidation temperatures (750 °C, 800 °C, 850 °C) were chosen to complete the fabrication. The influence of different oxidation temperatures on IrO_X_ electrode performance was studied by combining the pH sensing characteristics and the morphology surveys. In order to explain the reason for the pH electrode potential changing over time, X-ray photoelectron spectrascopy (XPS) was performed to analyze the changes in chemical composition and the elemental oxidation state of the electrode fabricated at 850 °C, both before and after one month of aging in distilled water. Results will be helpful for preparation of solid pH sensor for in-situ pH measurements of hardened concrete.

## 2. Experimental Program

### 2.1. Electrode Preparation

In this study, the carbonate melt oxidation method was used to fabricate the IrO_X_ electrodes. In this method, a cleaned iridium wires (Φ0.5 mm, 99.8% purity, 10 mm length, purchased from Alfa Aesar (Tianjin) Chemical Co., Ltd., Tianjin, China) were placed in a gold foil lined alumina crucible and covered with Li_2_CO_3_ powder. The crucible was heated in oven with a heating rate of 10 °C/min until the designed temperature (750 °C, 800 °C, 850 °C) was reached. The temperature was then maintained for 5 h under air atmosphere. After cooling down to room temperature, 1 M HCl solution was used to dissolve the solid carbonate, followed by washing the oxidized wires with distilled water and then drying them at 120 °C for 12 h. Finally, the end of the oxidized wire (about 1 mm in length) was scraped off and the oxidized wire was spot-welded to a copper wire. The contact area was then covered with epoxy.

### 2.2. Open-Circuit Potential Measurement

**Figure 1:**
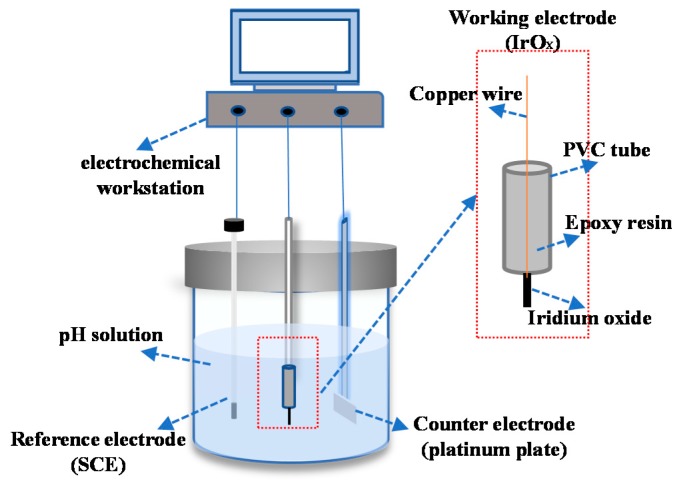
Schematic picture of the pH testing setup.

The pH response characteristic of IrO_X_ electrodes was examined by measuring the open circuit potentials in different test solutions. Three commercial pH buffer solutions with pH value of 4 (potassium hydrogen phthalate), 6.86 (mixed phosphate) and 9.18 (sodium tetraborate) were used to obtain a series of test solutions with different pH values (pH 1–13) by adding 1 M HCl or 1 M NaOH (all chemical reagents were purchased from Huadong Instrument, China). A glass pH electrode (E-201-C, INESA Scientific Instruments, China) was used to monitor the pH values of the test solutions. For each pH sensing points, we prepared a series of pH solutions and do pH testing one after another. Before each testing, the electrodes were rinsed with deionized water. The open circuit potential of the electrode was measured and recoded by an electrochemical workstation (VersaStat3, Princeton Applied Research, USA) with a platinum plate as the counter electrode and a saturated calomel electrode (SCE) as the reference electrode as shown in Figure 1. All potentiometric measurements were performed at room temperature (20 ± 3 °C), and the electrodes were kept in distilled water when they were not being tested.

### 2.3. Surface Analysis

Scanning electron microscopy (SEM) was performed with Quanta FEI 250. Atomic force microscopy (AFM) was conducted with Bruker Dimension Edge. The tests were conducted under ambient conditions with a scan size of 10 μm × 10 μm and scan rate is 10 μm/s. The silicon nitride tips (Bruker Company) and tapping mode were chosen in this study. X-ray photoelectron spectroscopy (XPS) was performed with Thermo ESCALAB 250XI by using the Al Kα (hν = 1486.6 eV) as its X-ray source. Xpspeak41 was used to process the data.

## 3. Results and Discussion

### 3.1. Surface Morphology

**Figure 2:**
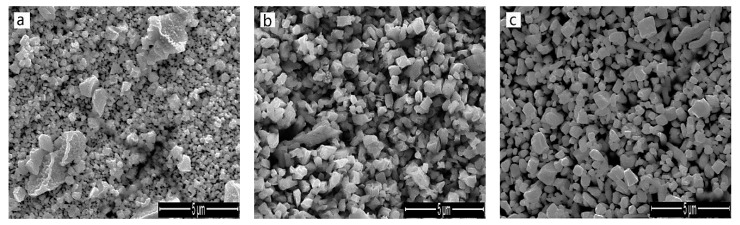
SEM images of IrO_X_ electrodes fabricated at 750 °C (**a**), 800 °C (**b**), and 850 °C (**c**).

The surface morphology of the IrO_X_ electrodes fabricated at different oxidation temperatures are shown in Figure 2. The films appear different from the iridium oxide films made by the thermal decomposition method, which usually reveals the mud-cracked morphology [34]. Electrodes made at 750 °C showed tiny cracks and uneven particle size and distribution. The films become more uniform as oxidation temperature increased. The iridium oxide films made at 850 °C had a dense and homogenous structure. The cracked and uneven layer leading to potential pathways to the electrode matrix may have been detrimental to the pH sensing. Furthermore, the iridium oxide film made at 750 °C fell off the substrate, while the film made at 800 °C and 850 °C stuck to the iridium substrate; they were hard to scrape off, even with a knife. This indicates that the surface morphology can affect the adhesion of the oxide film; the cracked and uneven film is less adherent than the dense, uniform film.

**Figure 3:**
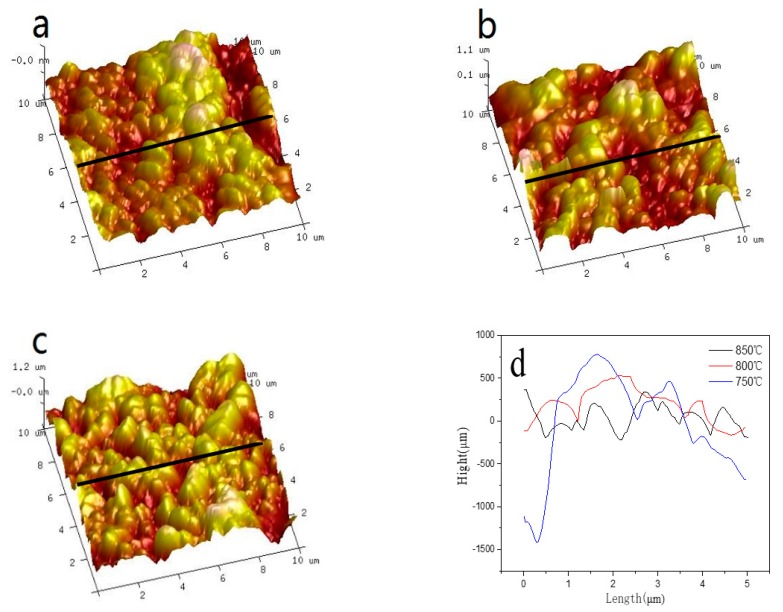
AFM images of IrO_X_ electrodes fabricated at 750 °C (**a**), 800 °C (**b**), 850 °C (**c**), and section roughness analysis (**d**).

Figure 3 shows the 3-D images of the surface of IrO_X_ electrodes obtained by AFM. Obviously, the particle size and distribution of iridium oxide film made at different temperatures are different. The section roughness analysis from the AFM results are shown in Figure 1d, from which it is clear that the particle size of IrO_X_ ranges from a few hundred nanometers to a few micros, and the morphology becomes more uniform with the increase in oxidation temperature.

### 3.2. Response Time

**Figure 4:**
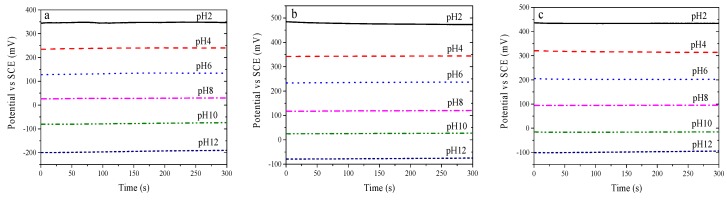
Response time, measured in different pH test solutions for IrO_X_ electrodes fabricated at 750 °C (**a**), 800 °C (**b**), and 850 °C (**c**).

pH electrode response time has yet to be universally defined, either in the literature or in product specifications, as the environmental conditions are different [35]. In this paper, the response time was defined as the time needed for a potential change rate of less than 1 mV/min [36]. The electrodes’ response time characteristics were evaluated by continuously measuring the open-circuit potential (Figure 4). Each of the electrodes obtained the stable potentials at each pH level. The average response times for electrodes made at 750 °C, 800 °C and 850 °C at different pH levels were 85 s, 28 s and 19 s, respectively. The electrodes made at 800 °C had slow response rates in strong acidic solutions. The response time of the electrodes made at 750 °C were longer than those of the electrodes made at 850 °C and 800 °C. This may due to the cracked and uneven iridium oxide film needing more time to react and equilibrate with the solution [37].

### 3.3. pH Response Characteristics

**Figure 5:**
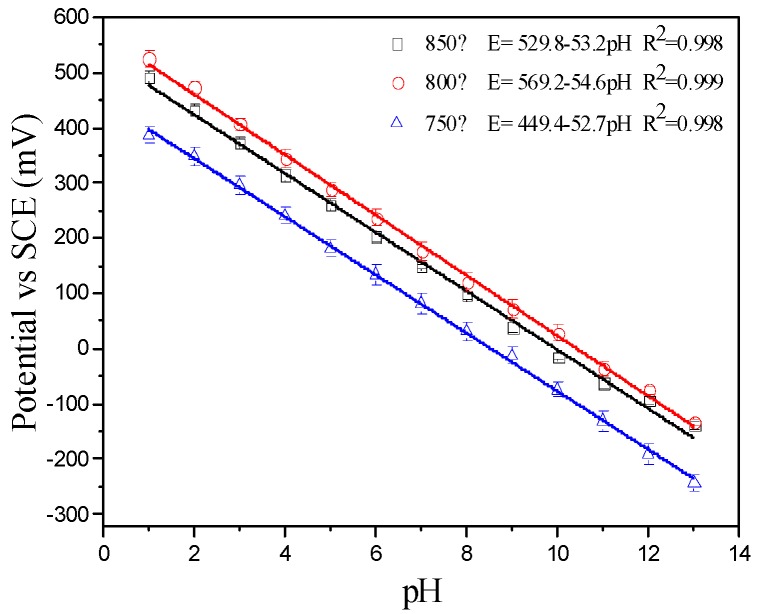
pH response characteristics of IrO_X_ electrodes fabricated at different oxidation temperatures.

The pH response characteristics of the IrO_X_ electrodes fabricated at different oxidation temperatures were investigated for the pH range of 1–13 (Figure 5). Results showed that the electrodes had similar sensitivity, −52.7 mV/pH, −54.6 mV/pH, and −53.2 mV/pH for oxidation temperatures of 750 °C, 800 °C, and 850 °C, respectively. These results corroborate the value obtained by Chen [32], which was slightly lower than the theoretical value of −59 mV/pH. The E-pH curves had a linear relationship with a correlation coefficient over 0.998.

### 3.4. Long-Term Stability

**Figure 6:**
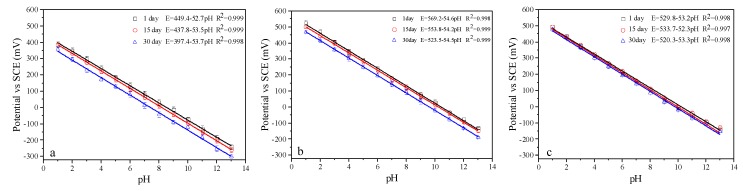
Long-term stability of IrO_X_ pH electrodes fabricated at 750 °C (**a**), 800 °C (**b**), and 850 °C (**c**).

The long-term stability of IrO_X_ pH electrodes were measured by calibrating the electrodes three times within one month. During this month, the electrodes were stored in distilled water. Results are shown in Figure 6. All of the calibration curves maintained linearity, and the slope varied no more than 1 mV/pH throughout the test period. However, the intercept (E^0^) decreased 52 mV, 45.7 mV, and 13.4 mV for the electrodes made at 750 °C, 800 °C, and 850 °C, respectively. This aging process, characterized by the decrease in E^0^ over time, has been observed by several researchers for different IrO_X_ electrode preparation methods [27,36,38,39,40]. Yao et al. [11] thought that the oxidation state and the hydration degree of the oxide film influence the long-term stability of the electrode.

### 3.5. XPS

**Figure 7:**
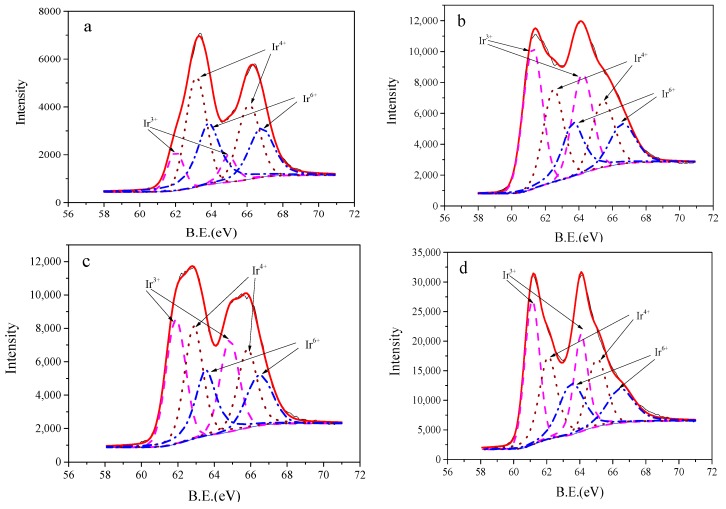
X-ray photoelectron spectra of Ir4f for IrO_X_ electrodes fabricated at 850 °C, (**a**) freshly prepared, (**b**) sample (**a**) after argon-ion etching for 90 s, (**c**) after aging for one month in distilled water, (**d**) sample (**c**) after argon-ion etching.

In order to further assess the potential drift mechanisms of the electrodes fabricated by the carbonate melt oxidation method, XPS were performed to investigate the chemical composition and element oxidation state change of the electrodes fabricated at 850 °C. This was done both before and after one month of aging. Deconvolution of Ir4f spectra was conducted considering the spin-obit doublet binding energy splitting of 2.95 eV. The peak ratio between Ir4f_7/2_ and Ir4f_5/2_ was constrained to 4:3. Several groups deconvoluted the Ir4f spectra, and an array of line shapes and interpretations of present species have been proposed [40,41,42,43,44,45]. In this investigation, the Ir4f spectra was deconvoluted into three species (Figure 7). The binding energies and the relative contents of different iridium species are presented in Table 1. The species present at 62 eV, 61.25 eV, 61.89 eV and 61.12 eV of Ir4f_7/2_ corresponded the existence of Ir^3+^ [40,45]. The species present at 63 eV, 62.49 eV, 62.87 eV, and 62.09 eV of Ir4f_7/2_ reflected the existence of Ir^4+^ [40,45]. The species present at 63.84 eV, 63.65 eV, 63.53 eV, and 63.5 eV of Ir4f_7/2_ reflected the existence of Ir^6+^ [46]. The table shows that the Ir4f binding energies attributed to Ir^3+^ and Ir^4+^ species on the electrode before argon-ion etching were significantly higher (0.75–0.77 eV) than the corresponding values after argon-ion etching. This phenomenon was probably due to the outer oxide layer having a higher hydration degree than the inner oxide layer [47]. The relative contents of the three iridium species show that for the freshly prepared electrodes, the Ir^4+^ and Ir^6+^ contents decreased by 14.6% and 16%, respectively. Meanwhile, the Ir^3+^ content increased by 30.6% after argon-ion etching. This indicated that the outer oxide layer had a higher average oxidation state than the inner oxide layer. This may have been due to the outer oxide layer covering the inner oxide layer, thus preventing the inner oxide layer from being further oxidized by the strong oxidizing species in melt carbonate. In contrast, for the electrodes that were aged for one month, the Ir^3+^, Ir^4+^ and Ir^6+^ contents were relatively stable, both before and after etching. The Ir^3+^ content also increased by 23.9%, while the Ir^4+^ and Ir^6+^ contents decreased by 10.9% and 13.3%, respectively, compared to the freshly prepared electrodes and the electrodes that had been aged for a month.

**Figure 8:**
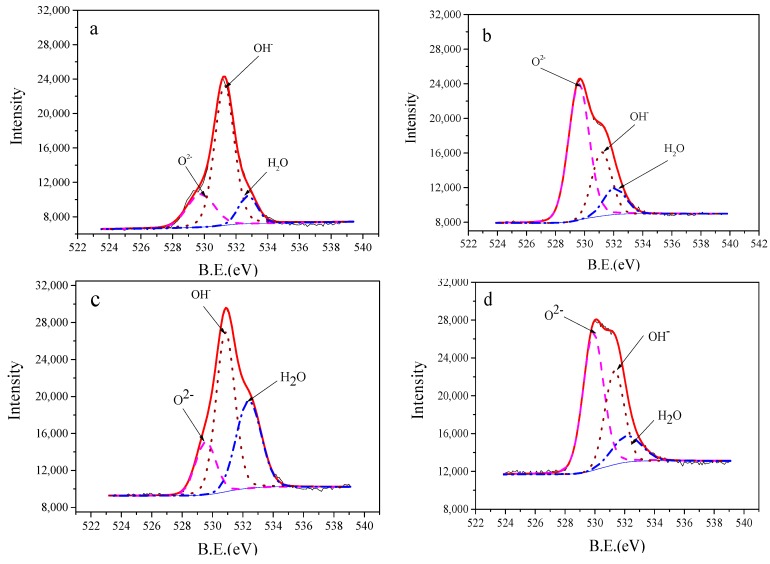
X-ray photoelectron spectra of O1s for IrO_X_ electrodes fabricated at 850 °C: (**a**) freshly prepared; (**b**) sample; (**a**) after argon-ion etching for 90 s: (**c**) after aging for one month in distilled water, (**d**) sample (**c**) after argon-ion etching for 90 s.

According to an earlier report on O1s deconvolution of IrO_X_ [40,41,47,48], three oxygen species may contribute to the intensity of O1s spectra. The range of binding energies for oxide is 530 ± 0.5 eV, for hydroxides it is 531.4 ± 0.5 eV, and for bound water it is 533 ± 1 eV. In our experiment, the deconvolution of O 1s spectra (Figure 8 and Table 2) shows three peaks. This indicates that the O1s spectra may contain all three kinds of oxygen. Analysis of the relative content of different oxygen species has found that the hydroxide species contributes most to the overall O1s signal of electrodes, both before and after one month of aging. However, the oxide (lattice oxidized O^2−^) became the major species after the ion etching process. This confirmed the different hydration degrees between the outer oxide layer and the inner oxide layer. It also indicated that the freshly prepared iridium oxide film was hydrated, due to the treatment of HCl solution and distilled water in the fabrication process.

The atomic ratios of Ir^3+^/Ir^4+^ and O1s/Ir4f in our experiment are summarized in Table 3. The Ir^3+^/Ir^4+^ atomic ratio increased 70% and O1s/Ir4f decreased 60% after one month of aging. This indicated that the Ir^3+^ complex species have an O/Ir ratio lower than that of the Ir^4+^ species. Huang et al. [40] have suggested that the pH response reaction is affected by the Ir^3+^/Ir^4+^ ratio and the hydration of the oxide film. Thus, the Ir^6+^ species have been regarded as the “defect structures”, and they have proposed the pH response reaction mechanism shown in Equations (1) and (2). In these equations, H_f_^+^ and H_s_^+^ are the H^+^ ions inside the hydrated oxide film and in the solution, respectively. Additionally, the Ir^3+^/Ir^4+^ ratio and the hydration of the oxide film can affect the value of the intercept, but cannot influence the sensitivity of the electrodes. This is consistent with the results of our experiment:

(1) $$\begin{array}{cc} 2\{[{\mathrm{IrO}}_{2}{\left(\mathrm{OH}\right)}_{2} & \cdot 2H_{2}{O]}^{2-}\cdot 2H_{f}^{+}\}+2e^{-}+2H_{s}^{+}\\ & ↔{\left[{\mathrm{Ir}}_{2}O_{3}{\left(\mathrm{OH}\right)}_{3}\cdot 3H_{2}O\right]}^{3-}\cdot 3H_{f}^{+}+3H_{2}O \end{array}$$

(2) $$\begin{array}{cc} E=E^{0} & +\frac{2.303\mathrm{RT}}{2F}\log\frac{\{[{\mathrm{IrO}}_{2}{\left(\mathrm{OH}\right)}_{2}\cdot 2H_{2}{O]}^{2-}\cdot {2H_{f}^{+}\}}^{2}\cdot {\left[H_{s}^{+}\right]}^{2}}{{\left[{\mathrm{Ir}}_{2}O_{3}{\left(\mathrm{OH}\right)}_{3}\cdot 3H_{2}O\right]}^{3-}\cdot 3H_{f}^{+}}\\ & \;=E^{0}+\frac{2.303\mathrm{RT}}{2F}\log\frac{{\{[{{\mathrm{IrO}}_{2}{\left(\mathrm{OH}\right)}_{2}\cdot 2H_{2}O]}^{2-}\cdot 2H_{f}^{+}\}}^{2}}{{\left[{\mathrm{Ir}}_{2}O_{3}{\left(\mathrm{OH}\right)}_{3}\cdot 3H_{2}O\right]}^{3-}\cdot 3H_{f}^{+}}\\ & \;+\frac{2.303\mathrm{RT}}{F}\log\left[H_{s}^{+}\right]=E^{0^{,}}-\frac{2.303\mathrm{RT}}{F}\mathrm{pH}=E^{0^{,}}-59.16\mathrm{pH} \end{array}$$

## 4. Conclusions

SEM and AFM images showed that the oxide film regularized in terms of particle size and distribution as the oxidation temperature increased. Sub-Nernstian responses of −52.7 mV/pH, −54.6 mV/pH, and −53.2 mV/pH have been obtained corresponding to 750 °C, 800 °C, and 850 °C, respectively. The better performance in response time and long-term stability was manifest in better quality oxide film made at 850 °C than at 800 °C or 750 °C. The pH response characteristics of the electrodes fabricated at the three different oxidation temperatures maintained linearity and the slope changed little, but the intercept decreased during the one-month aging process. The XPS results showed that the element Ir of the oxide film existed in the form of Ir ^3+^, Ir^4+^, and Ir^6+^. The relative contents of Ir^3+^ increased by 23.9%, while Ir^4+^ and Ir^6+^ decreased by 10.9% and 13.3% in the surface oxide film after one month of aging. An argon-ion etching experiment on the electrodes, both before and after one month of aging, resulted in the different hydration degrees along the depth of the oxide films. It also showed that the hydration effect occurred primarily on the surface of the oxide film.

## Figures and Tables

**Table 1:** Binding energies and relative contents of different iridium species, as observed from the Ir4f X-ray photoelectron spectra of electrodes fabricated at 850 °C.

Electrode	Species	Binding Ir4f_7/2_ (eV) Energies	Relative Content (%)
Freshly prepared	Ir^3+^	62	13.1
	Ir^4+^	63	46.3
	Ir^6+^	63.84	40.6
Freshly prepared and etched	Ir^3+^	61.25	43.7
	Ir^4+^	62.49	31.7
	Ir^6+^	63.65	24.6
Aged for one month	Ir^3+^	61.89	37
	Ir^4+^	62.87	35.4
	Ir^6+^	63.53	27.6
Aged for one month and etched	Ir^3+^	61.12	39.5
	Ir^4+^	62.09	33.2
	Ir^6+^	63.5	27.4

**Table 2:** Binding energies and relative contents of different oxygen species, as observed from the O 1s X-ray photoelectron spectra of electrodes fabricated at 850 °C.

Electrode	Species	Binding Energies of Ir4f_7/2_ (eV)	Relative Content (%)
Freshly prepared	Oxide	529.78	20.9
	Hydroxide	531.27	67.4
	H_2_O	532.74	11.7
Freshly prepared and etched	Oxide	529.6	62.1
	Hydroxide	531.17	25.8
	H_2_O	532.06	12.1
Aged for one month	Oxide	529.55	16.8
	Hydroxide	530.9	49.2
	H_2_O	532.74	34
Aged for one month and etched	Oxide	529.9	53.8
	Hydroxide	531.32	32.4
	H_2_O	532.15	13.8

**Table 3:** Atomic-ratio of Ir^3+^/Ir^4+^ and O1s/Ir4f.

Electrode	Ir^3+^/Ir^4+^	O1s/Ir4f
Freshly prepared	0.3	7.4
Freshly prepared and etched	1.4	3.9
Aged for one month	1	6.8
Aged for one month and etched	1.2	2.1
